# A Deep Learning Based Framework to Identify Undocumented Orphaned Oil and Gas Wells from Historical Maps: A Case Study for California and Oklahoma

**DOI:** 10.1021/acs.est.4c04413

**Published:** 2024-12-04

**Authors:** Fabio Ciulla, Andre Santos, Preston Jordan, Timothy Kneafsey, Sebastien C. Biraud, Charuleka Varadharajan

**Affiliations:** Earth and Environmental Sciences Area, Lawrence Berkeley National Laboratory, Berkeley, California 94720, United States

**Keywords:** oil and gas industry, undocumented orphaned wells, historical topographic maps, artificial intelligence, computer vision, semantic segmentation, U-net

## Abstract

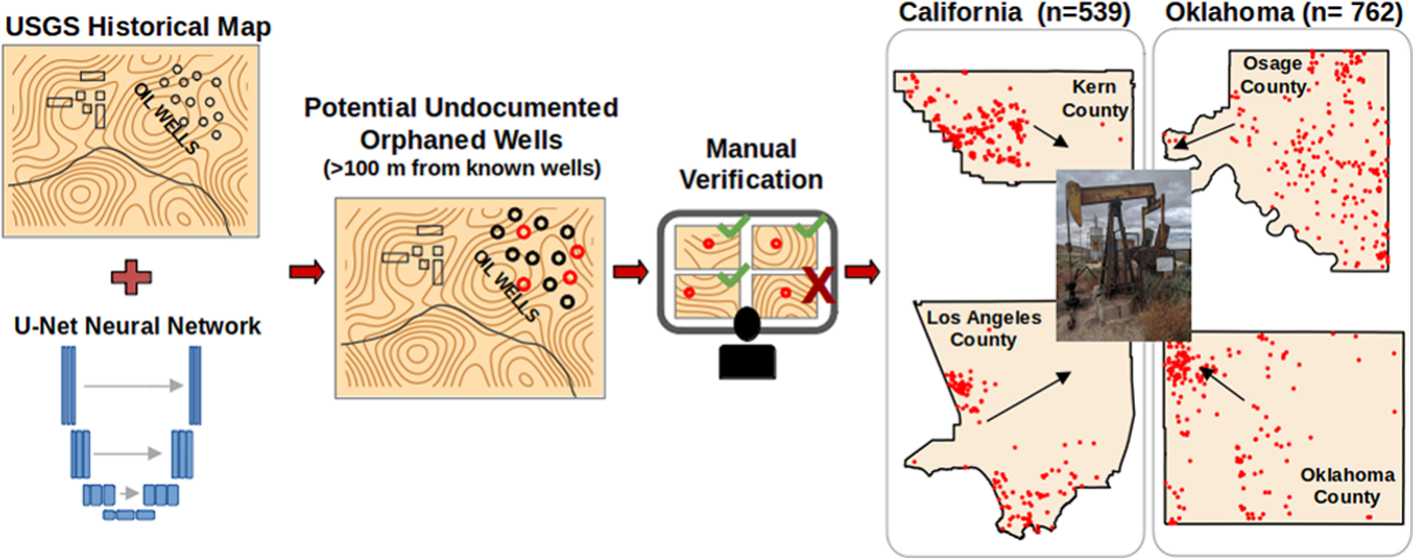

Undocumented Orphaned Wells (UOWs) are wells without an operator that have limited or no documentation with regulatory authorities. An estimated 310,000 to 800,000 UOWs exist in the United States (US), whose locations are largely unknown. These wells can potentially leak methane and other volatile organic compounds to the atmosphere, and contaminate groundwater. In this study, we developed a novel framework utilizing a state-of-the-art computer vision neural network model to identify the precise locations of potential UOWs. The U-Net model is trained to detect oil and gas well symbols in georeferenced historical topographic maps, and potential UOWs are identified as symbols that are further than 100 m from any documented well. A custom tool was developed to rapidly validate the potential UOW locations. We applied this framework to four counties in California and Oklahoma, leading to the discovery of 1301 potential UOWs across >40,000 km^2^. We confirmed the presence of 29 UOWs from satellite images and 15 UOWs from magnetic surveys in the field with a spatial accuracy on the order of 10 m. This framework can be scaled to identify potential UOWs across the US since the historical maps are available for the entire nation.

## Abstract 2

Thirteen hundred and one potential undocumented orphaned wells are identified in four counties in California and Oklahoma from historical topographic maps using a computer vision neural network.

## Introduction

The United States (US) has a long history of hydrocarbon extraction, with about 3.7 million oil and gas (O&G) wells drilled since the 1850s.^1^ Although abandonment requirements vary by state^2−6^ and may have differed in the past, today well operators are generally required to plug and abandon O&G wells when they are no longer profitably producing oil or gas. Wells that do not have “a legally or financially viable responsible party for plug and abandon operations,”^7^ such as due to bankruptcy, are considered orphaned.

An orphaned well may also be undocumented, defined by the Interstate Oil and Gas Compact Commission (IOGCC) as one “that is entirely unknown to the regulatory agency or a well of which the agency has some evidence, but which requires further records research or field investigation for verification”.^7^ Wells that are both undocumented and orphaned are referred to as Undocumented Orphaned Wells (UOWs). These mostly occur because the management, tracking and regulation of O&G wells in the US is overseen by state agencies, which were typically established many years after the first wells were drilled in their jurisdiction. Based on information provided by some states, the IOGCC estimates there are 310,000 to 800,000 UOWs in the US.^7^ Estimates of documented abandoned wells in the US vary from over 2.3 million^8^ to 3.6 million.^9^

Orphaned wells can present hazards to human health^10,11^ and ecosystems.^12^ These include the release of methane, a powerful greenhouse gas, hydrogen sulfide, and volatile organic compounds into the atmosphere,^13^ as well as contamination of freshwater aquifers and surface water^14^ from oil, brine and methane leakage.^15,16^ Notably, methane leakage from orphaned wells is a potentially significant source of carbon emissions to the atmosphere during oil and gas operations.^11^ Hence, identification of UOWs will allow proper characterization and plugging to mitigate potential environmental risks.

Throughout the years, several methods have been developed to find UOWs, typically at spatial scales ranging from small plots to oil fields. Observational approaches leverage ground-based or remote sensing surveys using satellite imagery, LiDAR, magnetometers and other instrumentation, such as gas sensors or metal detectors, to identify the precise location of the UOW.^17−20^ Data mining approaches, instead, include searching for well locations from O&G databases, historical records, photographs, and lease or farmline maps.^21^ UOWs have also been reported to state agencies when discovered by individuals or citizen science efforts.^22,23^

To date no methods have been developed to identify the precise location of UOWs across whole states and basins. It has been difficult to scale up and transfer estimates from local studies to other regions due to the diversity of landscapes, oil production histories and practices, and the amount of documentation available in different parts of the US. In 2022, the Consortium Advancing Technology for Assessment of Lost Oil & Gas Wells (CATALOG)^24^ program was initiated by the US Department of Energy to assist regulatory agencies in reducing the impact of UOWs. One of the program’s primary goals is to develop methods to identify the locations of UOWs across the US.

In this study, we describe a semiautomated, transferable method developed for the CATALOG program to identify UOWs at regional scales in the US using the US Geological Survey (USGS) Historical Topographic Maps Collection (HTMC), a digital archive of about 190,000 georeferenced topographic maps published between 1884 and 2006.^25^ We focus on a subset of these maps, referred to as “quadrangles”, which have consistent symbols for a variety of natural and manmade features, including O&G wells. There are about 131,000 quadrangles in the HTMC, comprising 69% of the entire database.

Traditional approaches for image feature extraction involve techniques like edge detection, color separation using clustering, or template matching to identify regions of interest. Many studies have used this approach on historical maps for the identification of topographical lines,^26^ elevation spots,^27^ roads^28^ or other features.^29^ The main advantage of these techniques is that they do not require training data, but their performance is sensitive to the choice of parameters. For this reason it is difficult to find a unique set of parameters that apply across the variability in map backgrounds and color distortion occurring in the quadrangles, despite their focus on consistency.

In recent years, neural networks have proven to be extremely effective in the field of computer vision, often outperforming traditional approaches in tasks like image segmentation. In particular, the convolutional neural network U-Net model^30^ is a popular choice due to its skill in diverse image identification tasks. Contrary to traditional computer vision algorithms the U-Net model is capable of generalization and does not require parameter tuning for different maps.

The objective of this study is to demonstrate that the HTMC quadrangles are a good source of information for identifying UOWs. We accomplish this by developing a semiautomated workflow that can quickly detect wells in the maps, and verifying the results using field surveys and satellite images. Hence, we developed a framework that (1) identifies O&G well symbols from the HTMC with high precision leveraging the state-of-the-art U-Net computer vision algorithm, (2) classifies potential UOWs by screening against O&G databases, and (3) provides the ability to verify the results quickly through a custom script.

We demonstrate the effectiveness of this framework by identifying 1301 potential UOW locations in the major oil producing regions of Kern and Los Angeles counties in California, and Oklahoma and Osage counties in Oklahoma. For some sites we provide verification of the presence of UOWs based on evidence from satellite images and field investigations, and find that our algorithm can identify UOW locations with an average accuracy of the order of 10 m. The method can eventually be applied to other maps, besides the quadrangles, that lack consistency in features and symbols, although this would require identifying and labeling all the variations across maps. To our knowledge, this is the first approach developed to identify UOWs at county scales, enabling stakeholders to rapidly identify potential UOW locations in their regions of interest and prioritize them for field verification.

## Methods

### Data Sets

Maps from the HTMC are digitally available for the contiguous US (CONUS), Hawaii and part of Alaska as georeferenced raster images,^31^ where each pixel, and by extension a feature in the map, is associated with specific geographical coordinates. Within the HTMC, we used a series of maps issued between 1947 and 1992 covering the CONUS, each spanning 7.5 min of longitude and latitude at 1:24,000 scale, referred to as “quadrangles”. These maps are useful for identification of UOWs over large areas because they use consistent colors and symbols to indicate natural and man-made features such as mountain tops (represented as brown crosses), rivers and canals (blue lines), vegetated areas (various green patterns), roads (black double lines), buildings (black rectangles), water tanks (filled black circles), and importantly O&G wells (hollow black circles)^32^ (see Figure SI1 for examples of maps used in this study).

The records of documented O&G wells were retrieved from official state databases. For California, we used the California Geologic Energy Management Division (CalGEM) database containing information for 241,684 wells.^33^ In Oklahoma we used 462,445 records from the Oklahoma Corporation Commission^34^ and 43,822 records from the Osage Bureau of Indian Affairs^35^ for a total of 506,267 wells.

### Selection of Study Areas

To demonstrate our workflow, we used counties, rather than oil fields or other geographic domains, as spatial units. Counties have clearly defined boundaries, and include areas beyond oil fields that have a greater variety of land uses and topographies, which prevents bias toward regions of predominant hydrocarbon extraction. We used quadrangle maps that covered any part of a county, resulting in study areas that are slightly larger than official county boundaries.

We first identified states of interest as the ones with substantial early oil production as noted from annual production data available for all states in the US through 1935.^36^ California and Oklahoma were interchangeably first and second for annual oil production in the first decades of the 1900s (Figure SI2a). California had the most cumulative production from 1914 to 1935, and Oklahoma was the second largest from 1917 to 1933 (Figure SI2b). Thus, we chose to investigate areas in California and Oklahoma, because of the relevance in early oil production of these two states.

Within each state of interest, we repeated the same analysis at a finer scale to select counties with the highest oil production in the early years. We did not consider dry gas production because it was not a target of early hydrocarbon resource development. In California, Los Angeles County had the largest annual production prior to 1900 and Kern County thereafter (Figure SI3a),^37^ and the two counties had the most cumulative production since 1901 (Figure SI3b). In Oklahoma, Osage County has a portion of the oldest and largest oil field in the state (Bartlesville-Dewey, discovered in 1897) and almost the entirety of the fourth largest field, also discovered relatively early (Burbank, discovered in 1920). Oklahoma County includes most of the second largest field, discovered relatively early (Oklahoma City, discovered 1928), and much of another large field (Edmund West, discovered 1943).^38^

In summary, we chose to apply our workflow to Kern and Los Angeles counties in California, and Osage and Oklahoma counties in Oklahoma because of their production history, as well as distinct land use (rural and urban) to test the performance of the algorithm across a diversity of map types. The two states also had gaps between the start of oil production and the establishment of well documentation regulations. The first oil and gas regulatory agency in California, the Division of Oil, Gas, and Geothermal Resources, now known as CalGEM, was established and started regulating the industry in 1915.^39,40^ In Oklahoma, the Oklahoma Corporation Commission was established in 1907 and started to fully regulate the oil and gas industry in 1915.^41,42^ We also considered these counties as locations where field activities were planned as part of the CATALOG program, to enable on-the-ground confirmation of potential UOWs detected from our algorithm.

### Labeling and Preprocessing

**Figure 1:**
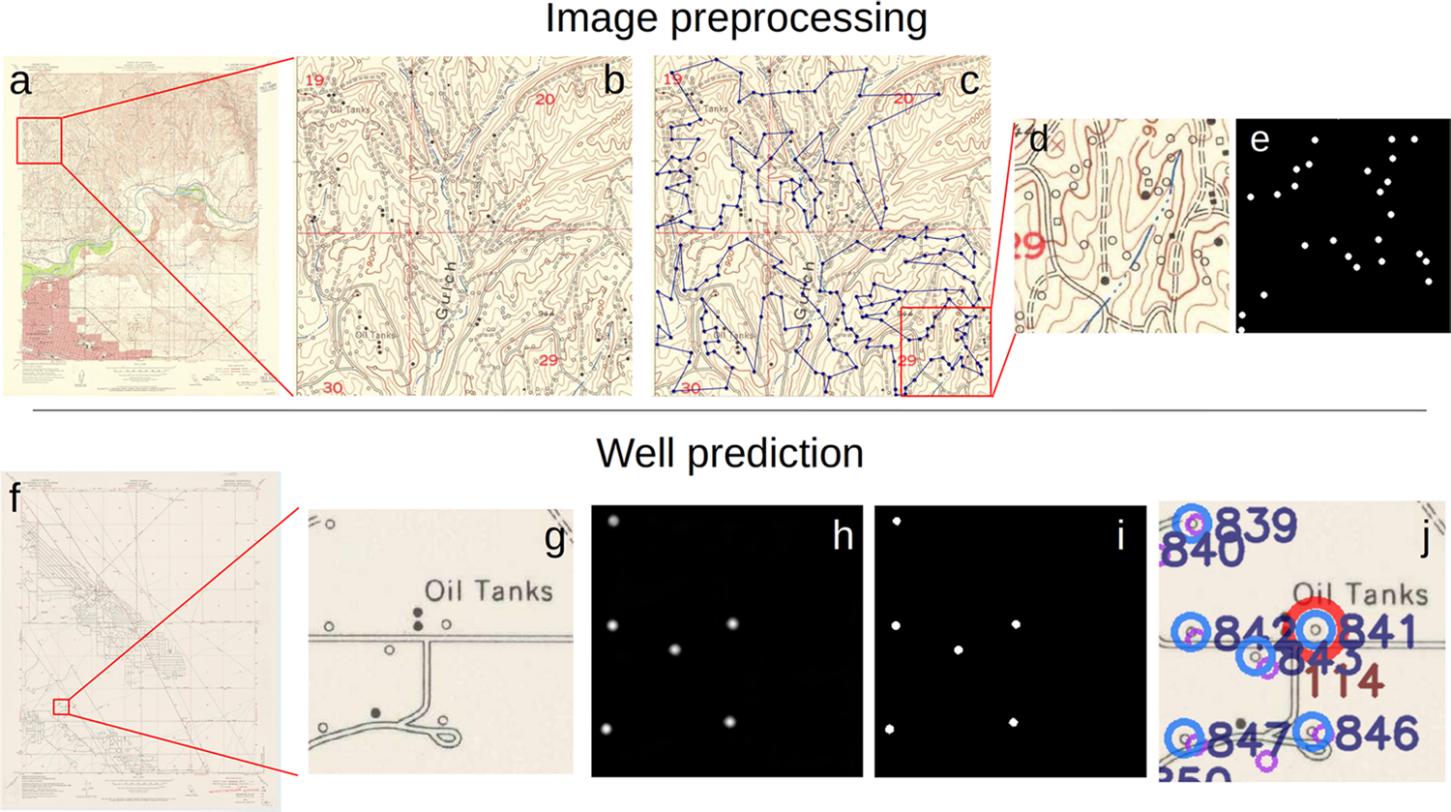
(a–e) Visual workflow of pretraining processing steps using (a) a 1954 map of Bakersfield, CA, (b) a 1000 × 1000 pixel tile within it, (c) the locations of the labeled well symbols displayed by blue dots connected by lines, (d) a 256 × 256 pixel inset and, (e) its relative binary mask generated from the labels to be used as pairs during training. (f–j) Visual workflow of the well detection process using (f) a 1953 map of Belridge, CA, (g) a selected 256 × 256 tile within it showing oil wells and tanks as hollow and solid black circles respectively, (h) the probability map of well pixels as predicted by the computer vision algorithm, (i) the final identification and localization of wells, (j) the visualization of the detected wells on a map shown as blue circles and unique identifiers. Documented wells are shown in purple, and a potential UOW in red, with the distance to the closest documented well displayed in meters (brown number).

We selected 79 maps in California, representative of the different background colors and landscape features present in the HTMC (Figures 1a and SI1). From each of these maps we cropped one or more 1000 × 1000 pixel tiles (Figure 1b), where each pixel is approximately 2 m ground resolution, for a total of 440 tiles. We used the software Labelme^43^ to manually tag the locations of 11,046 wells by visually identifying well symbols in the map and recording the location of their centers (Figure 1c). It took a total of 40 h for a single operator to complete this task. For areas where no wells are present, no locations were recorded. We intentionally did not use maps from Oklahoma for training to demonstrate the generalizability of the workflow to new regions that the model was not trained on. This leverages the consistency in quadrangle maps throughout the United States, and would avoid the need to create labels for every potential region where UOWs may exist.

After the labeling, we generated a corresponding binary mask for each tile, where the location of a well is denoted by a solid disc of 4 pixels radius (and area of 49 pixels) with value 1. Tiles with no wells have masks with only 0 values. Because the well symbol size varies throughout the map series, averaging about 6 pixels in radius, we chose slightly smaller discs of 4-pixel radius to ensure that each disc is entirely contained within the hollow black circles, accounting for their size differences.

Each 1000 × 1000 pixel tile and its corresponding mask were split into 16 equally spaced 256 × 256 pixel tiles for a total of 7040 image/mask pairs (Figure 1d,e). Every pair is augmented 10 times using a random rotation ranging from 0 to 360°, and random horizontal and/or vertical flip, ultimately producing 70,400 image/mask pairs. This constitutes the data set for our detection algorithm, which was randomly split 60–20–20 for training, validation and test sets. To prevent data leakage, no samples from the same original map were allowed to be present in more than one set. Also, labeled map tiles are categorized according to their dominant land cover color background, i.e., red for urban areas, green for vegetation, blue for water bodies and white for undefined, and each category is represented in each set of the model. The validation set was used to tune the model parameters and the test set constitutes out-of-sample images used solely for performance evaluation.

### Segmentation Model and Training

We used a U-Net neural network,^30^ a state-of-the-art deep learning computer vision model, to detect well symbols on the maps. The model performs image segmentation using pairs of images and their relative binary masks as inputs, and is trained to identify pixels of value 1 in masks as target objects (see Supporting Information for details on model implementation for this study). To evaluate the performance of the model, we used the intersection over union (IoU) metric, which quantifies the ratio of correctly detected pixels.^44^ It took about 2 h to complete the training using 4 GPUs in parallel when using an exclusive node on the Department of Energy’s NERSC supercomputer.

### Segmentation Postprocessing

The outcome of the image segmentation is a probabilistic map with pixel values ranging from 0 to 1, with values closer to 1 indicating the presence of a well (Figure 1h). Pixels with values equal to or greater than 0.5 were mapped into ones, while values below 0.5 were mapped into zeros (Figure 1i). Pixels with values 1 and less than 4 pixels apart (i.e., the radius of well masks) were considered contiguous and grouped into one object, and their centroid computed as an average of the object’s pixel coordinates. For evaluation purposes, if a centroid was located up to 4 pixels away from the center of a well symbol identified during the labeling process, the detected object was considered a true positive (TP). Conversely, if the centroid of the detected object was more than 4 pixels apart from the center of any well symbol, the object was considered a false positive (FP) for the purpose of the detection by the algorithm. Finally, hollow black circles present in the map that were not detected by the model were considered false negatives (FN). The precision, defined as TP/(TP + FP), and recall, defined as TP/(TP + FN), were tuned by thresholding the detected objects by their area. The precision and recall in the validation set were equal to 0.99 and 0.88 respectively, when the threshold area was set to 45 pixels (see the Supporting Information for details about the threshold choices).

### Workflow to Identify Potential UOW Locations in Topographical Maps

After training and validating the neural network, we identified potential UOWs by the following process (Figure 1f–j). First the map margins were stripped and the resulting image sliced into 256 × 256 tiles (Figure 1g) with a 25-pixel overlap with adjacent tiles to ensure that any well symbol cut off during the slicing was present as full circles in one of the tiles. The model was applied to each tile separately, resulting in probabilistic maps (Figure 1h) that were combined using a union operator and transformed into binary masks (Figure 1i), where pixels with values 1 are grouped as described above. Finally, we used the projection information present in the georeferencing metadata of each map to translate the pixel location of the detected wells into geographical coordinates. These steps resulted in a list of locations of the O&G symbols (i.e., hollow black circles) present in each map.

We then matched the geographical locations of wells detected by the model with documented wells from state databases (Figure 1j). We included all the documented wells present in a database, irrespective of their status (active, plugged, etc.) or spud date. In particular, we did not use spud dates as a filter because they are only available for a subset of documented wells across our study areas.

If a detected well was located more than 100 m away from any documented well, it was flagged as an “unvetted potential UOW”. The choice of this value is justified by the fact that documented wells can have errors in location coordinates on the order of tens of meters, particularly for wells that predated modern GPS technology,^21^ or due to errors in the georeferencing process. Also, we estimated a 15 m uncertainty in the locations of potential UOWs based on differences in coordinates of the same well in multiple colocated maps issued at different time periods.

We then visually inspected the corresponding well symbols of all unvetted potential UOWs, and only retained those that are confirmed to be hollow black circles. To do this at scale, we developed a custom script that isolates and displays the area surrounding each unvetted potential UOW, enabling rapid manual confirmation with a simple mouse click. In this way, we filtered out incorrectly detected symbols and produced a list of vetted potential UOWs (Figure SI4).

Since the quadrangle maps are published within a 45 year time range, multiple maps can cover the same geographical domain at different time periods. Hence, we removed redundancies by merging wells detected in two or more maps issued at different time periods that are less than 15 m away from each other. The merge distance of 15 m is based on the average distance determined by visual inspection of 50 well locations displayed in at least two maps at different times. The final outcome of this workflow was an atemporal location of unique potential UOWs, which we refer to as potential UOWs.

### Verification of Potential UOWs with Satellite Imagery and Historical Photographs

We used modern satellite images from Google Earth to find visual evidence of the presence of a well. Since the spatial resolution of the satellite images does not allow for direct identification of a wellhead on the surface, we visually inspected the area surrounding the location of a potential UOW (at maximum zoom) for the presence of a well-related structure. In particular, the detection of an oil rig was used as a proxy for the presence of a well. Other structures, like an oil pad, storage tanks and disturbed terrain, were considered supporting evidence.

We noted the locations of documented wells surrounding each UOW to avoid mistakenly identifying them as potential UOWs. The presence of visible well-specific structures within 100 m (matching the buffer radius in the potential UOW detection methodology) are used as evidence to confirm the presence of the UOW. The approximate center of the visible feature was recorded as the actual geographical coordinates of the well, and used to compute the distance between the observed UOW site and the location detected by our algorithm.

Since wells can be cut off below the surface during plugging and abandonment, there could be many instances where UOWs do not have visual evidence in current satellite imagery. For this reason, we used historical aerial photos, taken between 1927 and 2012 from the geospatial collection of the University of California Santa Barbara (UCSB) library^45^ to identify historical infrastructure. For a few potential UOWs, we downloaded older aerial photos (available at no cost) of the corresponding region, dated as close as possible to the publication date of the topographic map showing the potential UOW. Since the photos were not georeferenced, we manually georeferenced each photo using features such as roads to match the map features. We prioritized field investigation in sites where a historical aerial photo suggested the presence of a well, indicated by visible features such as storage tanks, wells, derricks, and ancillary equipment for field examination.

### Field Verification of Potential UOWs

Field surveys were used to verify the locations of some potential UOWs. The presence of a well in the field was verified by the detection of a magnetic anomaly consistent with the presence of a quasi-vertical metal pipe below ground. The equipment used consisted of a backpack-mounted Geometrics G-864 magnetometer with cesium vapor technology to measure the total magnetic field, paired with a nonmagnetic Tallysman GPS to simultaneously collect locations, and a Getac ZX70 tablet to observe the magnetic measurements in real time. In exploratory field campaigns, we also used a Dunham & Morrow DML2000-XR portable metal detector to identify the presence of buried metal structures. The detailed field data collection workflow is described in the Supporting Information.

## Results

### Identification of UOWs from Historical Topographical Maps Using Deep Learning

Our computer vision model, tested on 14,080 data points that were not present in the training set, provided a precision of 0.98 and recall of 0.88, which reflects our conservative choice of model parameters to favor higher precision over recall. The ultimate performance of our workflow, as measured by the ratio of vetted to unvetted potential UOWs (hereafter referred to as RVU), varied from 30 to 98% depending on the study area (Table 1). An explanation on the reduction of performance from the algorithmic precision to the RVU is presented in the Discussion and Supporting Information.

**Table 1:** Summary of the Wells Identified in the Four Counties in California and Oklahoma Chosen for This Study^a^

county	Kern, CA	Los Angeles, CA	Osage, OK	Oklahoma, OK
oldest field discovery	ca. 1890^46^	1876^47^	1904^b^^38^	1928^b^^38^
production first regulated	1915	1915	1915	1915
surface (km^2^)	21,140	12,310	5,970	1,860
total maps	564	513	96	60
maps per 1000 km^2^	26.7	41.7	16.1	32.3
documented wells	156,445	23,034	43,962	6,314
detected wells	58,892	42,975	13,755	3,675
unvetted potential UOWs	959	1,518	552	519
vetted potential UOWs	748	462	543	401
vetted to unvetted ratio (RVU)	0.78	0.30	0.98	0.77
potential UOWs	298	181	261	204
potential UOWs per 1000 km^2^	14.1	14.7	43.7	109.7
potential UOWs to documented wells ratio	1.9 × 10^–3^	7.9 × 10^–3^	5.9 × 10^–3^	3.2 × 10^–2^

a Well counts in this table only include those present within county lines. An additional 357 potential UOWs (60 in California and 297 in Oklahoma) were identified in surrounding regions outside county lines present in corresponding HTMC quadrangles.

b Oldest >100 MMbbl.

Using our workflow, we identified the unique locations of a total of 1301 potential UOWs (Table 1), of which 539 are in California (14 UOWs/1000 km^2^ in Kern and Los Angeles counties), and 762 are in Oklahoma (43 UOWs/1000 km^2^ in Osage County and 110 UOWs/1000 km^2^ in Oklahoma County). The coordinates of each well are provided as tables and geospatial files in an associated data set in the Supporting Information.

The counts of potential UOWs identified using our method are likely underestimated for various reasons, and the identification of potential UOWs can be affected by errors in documented well locations (see Discussion section for details). Additional verification from both visual inspection of the topographic maps and evidence from field campaigns or satellite images are required to confirm UOW locations and estimates.

### Verification and Prioritization Using Satellite or Aerial Imagery

**Figure 2:**
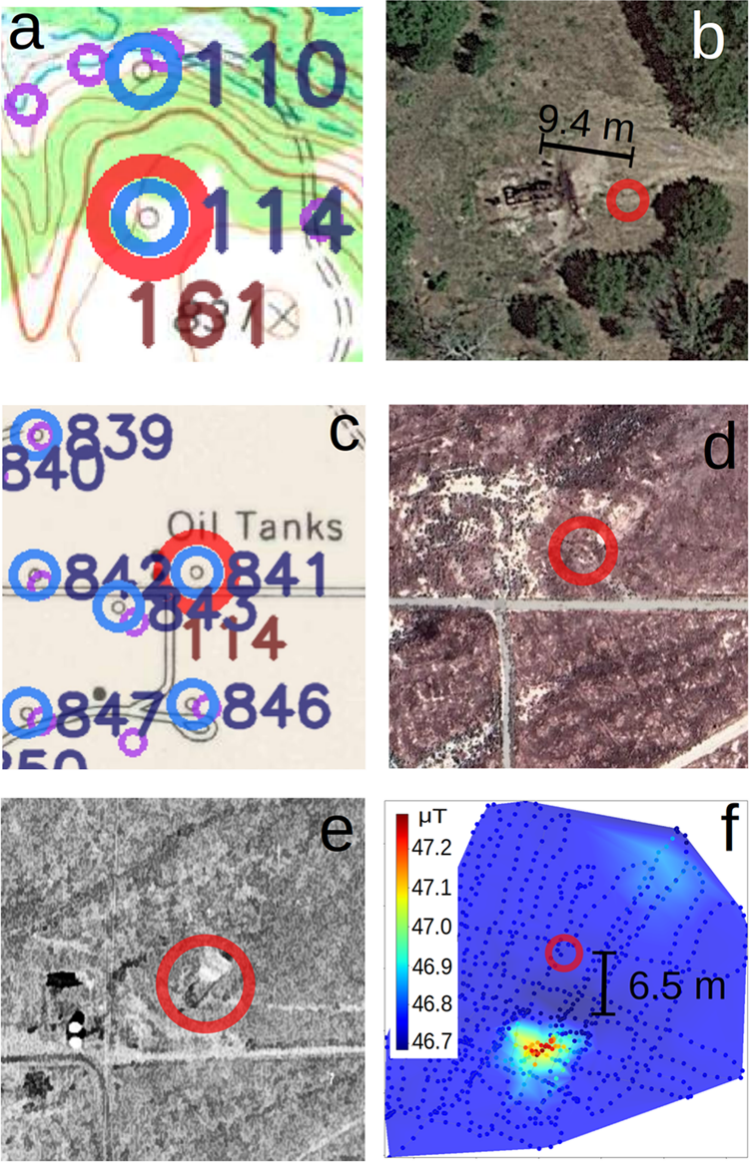
(a) Portion of an HTMC map in Osage County, OK from 1973. Colored circles indicate O&G wells: purple = documented wells, blue = detected wells, and red = potential UOWs. The numbers in dark blue are unique IDs assigned to each detected well. Numbers in brown represent the distance in meters of the potential UOW to the closest documented well. (b) Zoomed satellite image of the area showing an oil rig 9.4 m away from the detected well symbol. (c) Portion of an HTMC map for Kern County, CA from 1953 with wells indicated as described in (a). (d) Satellite image of the same area of (c) showing no structure currently visible. (e) Zoomed-in historic aerial photo from 1956 showing the presence of a structure. (f) Magnetic survey (dots) showing signal compatible with the presence of an underground well. Maximum value of the anomaly being 6.5 m away from detected coordinates. The background is a 2D linear interpolation of the magnetic field from the dots on a color scale shown on the right, and is intended as a visual aid to visualize the magnetic field anomaly.

In Oklahoma, several potential UOW locations are identifiable from visual inspection of Google Earth satellite images (Figure 2a,b). After inspecting all the 261 locations of potential UOWs within the borders of Osage County, we found 29 sites that had clear evidence of the presence of well-specific structures in the vicinity of the potential UOWs (Table SI1 and Figure SI5). This suggests that most of the potential UOWs in this region have no above ground structure visible from a satellite or that they are buried underground. For the 29 sites with visible surface features, the center of the oil rig was considered to be the location of the well. The average distance between the locations of the potential UOWs detected using our algorithm and the sites identified by satellite imagery is 9.4 ± 0.9 m. Although the exact location of a wellhead is typically at one end of a pump jack, choosing the center of the rig does not substantially affect the average distance between the satellite-based estimate and model-generated coordinates, when considering multiple wells.

In contrast, in California many of the locations did not have surface features detectable by satellite imagery since wells are typically cut off below the surface during plugging and abandonment (e.g., Figure 2d). For this reason we visually investigated 50 potential UOW sites identified by our algorithm in Kern County using aerial photos from^45^ and found that 25 sites had evidence of oil extraction activity such as storage tanks, derricks and ancillary equipment (Figure 2e). Because of their low resolution, the images cannot be used to confirm the presence of a well. Instead, the sites with photographic evidence of historical production activities were prioritized for further field investigation.

### Field Verification of UOW Locations

In June 2023, we conducted an exploratory field campaign to determine the locations of 21 detected potential UOWs in Kern County (Table SI2). We found that 8 of the sites were inaccessible (i.e., on private property). Additionally, we found that the portable metal detector was not reliable enough to be used as a tool to confirm the presence of UOWs due to the low signal-to-noise ratio, potentially caused by other metal structures (e.g., pipes or construction materials) in the vicinity of well structures. In February 2024, we conducted a second field campaign in the same area of Kern County to visit the 13 accessible sites. Using the backpack-mounted magnetometer, we confirmed the presence of 9 UOWs based on the magnetic anomalies compatible with the existence of a buried vertical metal pipe (Figure 2f and Table SI2 and Figure SI6a,b). In March 2024, we conducted another field campaign in Osage County to visit the locations of 14 potential UOWs (Table SI2), of which 6 were confirmed as UOWs using the magnetometer survey (Figure SI6c,d). No magnetic anomalies were detected for the remaining 8 wells that were all located in the same area. A visual representation of the field verification workflow can be found in Figure SI7.

Overall we found magnetic anomalies compatible with the presence of wells in 15 out of 27 sites. The wells detected were on average 11.7 ± 1.8 m from the coordinates of potential UOWs identified using our workflow, which is statistically equal to the average distance found using satellite imagery considering their respective standard errors.

## Discussion

### Accuracy of Potential UOW Location Estimates

In this study, we demonstrated a workflow that applies a deep learning computer vision algorithm to historical topographic maps to precisely locate potential UOWs. Through this workflow, we found the locations of 1301 potential UOWs in four US counties with long oil production histories. We confirmed the accuracy of the geographical coordinates to be on the order of 10 m through satellite image analysis and field investigations, which indicates that the results from our algorithm can be reliably used to discover UOWs.

However, the counts of potential UOWs identified through this workflow are likely underestimated for various reasons. First, these estimates are derived from quadrangle maps, which were issued between 1947 and 1992 that may not contain wells that had already been buried at the time of mapping. Also, the number of maps covering the same area throughout these years can vary, with some locations having very limited temporal coverage. As a result, wells built and dismantled in between the publication of two consecutive maps in the same area may not be detected through our workflow. Additionally, merging wells present in the same location across maps issued at different time using a buffer radius (see Methods section) can also result in underestimates of well counts.

Second, our definition of a UOW is based on the choice of a 100 m spatial buffer between detected and documented wells. Despite this being a reasonably large buffer, we found that some of our potential UOWs may actually be documented wells because of location errors in the official databases or topographic maps. For example, in our field investigations, we found plaques with lease and well names for five of the six confirmed potential UOWs in Osage County. No documented wells are present in the official database located within 100 m from these potential UOWs but some potential matches to known wells can be hypothesized by comparing leases and names (API numbers are not always available). Thus, our work flow also provides a means to improve the accuracy of well locations in official databases.

Third, when finding a match for potential UOWs in the official state databases we search across all the documented wells irrespective of their spud date, including those drilled after the historical maps were issued. Ideally, documented wells that had spud dates subsequent to the issue date of a map would be excluded in the comparison, which could result in a larger number of potential UOWs being discovered. However, we did not do this since only 32% of wells in California and no wells in Oklahoma had spud dates in the state databases.

Finally, we acknowledge that despite the manual vetting process, human identification errors of the black circles can occur due to the quality and resolution of some of the well symbols on the quadrangles, particularly when these are indistinguishable to the human eye from other similar features (e.g., black squares indicating buildings and blue circles denoting water wells). Hence, we emphasize that subsequent to identification of a potential UOW from our workflow, confirmation through of remote sensing imagery or field investigations is required to verify the presence of the well.

### UOW Identification Enabled by Recent Advances in Deep Learning and Computer Vision

The quadrangle maps within the HTMC are the most consistent series of georeferenced, historical maps with continental-scale coverage that we are aware of. This makes it possible to use automated computer vision approaches, which rely on the same graphical data for feature extraction. However, detection of O&G well symbols presents nontrivial challenges, even with the consistency in features across the quadrangles. First, the digital HTMC maps were generated by scanning paper maps present in archives and libraries. They have significant distortions in color due to the printing and scanning process, as well as the natural discoloration in the physical maps after decades of use. Hence the maps can contain distinct color patterns, which are particularly evident in the background colors that represent different land cover types (Figure SI1). The color distortion also affects the quality of the map symbols, causing variation in colors and size throughout the data set. Thus, it is not possible to precisely determine the size of the well symbols because of variations in their radii across maps. In some cases, even the human eye cannot distinguish between variations such as the blue and black colors used to represent water and O&G wells, respectively.

Traditional computer vision approaches, such as edge detection and template matching, cannot generalize across the variations present in the quadrangle maps. However, modern neural networks for computer vision are capable of generalization and do not require parameter tuning to perform proficiently in contexts different than the ones they have originally been trained. In particular, the U-Net model is a convolutional neural network that performs semantic segmentation for pixel-level classification, and is trained on the Imagenet data set. An alternate deep learning approach would involve object detection with models such as YOLO.^48^ Comparisons between these two techniques are limited, but two recent studies suggest that the U-Net outperforms YOLO for the tasks investigated.^49,50^ As described below, we leverage the circular symmetry present in the well symbols by tagging their centers in the labeling process. This allowed us to translate the symbol detection task into a semantic segmentation one in an efficient manner, and to use the better performing U-Net model. When fine-tuned on our map training set, the U-Net model also outperforms traditional computer vision approaches (see Supporting Information and Figure SI8 for details). For example, this model is less prone to mistakenly identify shapes of comparable size (e.g., squares versus circles).

Our two-part workflow for identifying UOWs first involves using the U-Net model to detect well symbols in a map, and a second step where the detected symbols are classified as potential UOWs based on their proximity to documented wells. The low values of the ratios of potential UOWs to documented wells (Table 1) indicate that identification of UOWs is computationally an imbalanced learning problem, with an extremely small number of targets to identify from the entire data set. This has important implications for the overall performance of our workflow, requiring extremely high algorithmic precision to identify the small number of desired targets. Hence, the minimization of prediction errors through improvement of precision is important, since any misclassification error is compounded by the imbalance of potential UOWs to documented wells. We aimed to achieve this goal by (1) utilizing a consistent, high-resolution geospatial data set containing information about O&G wells across multiple regions, and (2) developing an algorithm emphasized on high precision for detection of well locations. In particular, we chose to tune one of the few free parameters of the U-Net model, namely the size above which a detected object is considered a correctly identified symbol (referred to as the area threshold), to deliberately favor precision over recall (see details of U-Net architecture in the Supporting Information). This was done to minimize the number of FPs (requiring higher precision) instead of maximizing the overall number of identified objects (resulting in lower recall).

However, improving model precision alone is not sufficient to result in high performance of the entire workflow. For example, our model detects wells with a precision of 0.98, while the ratio between vetted to unvetted UOWs (RVUs) range from 0.3 to 0.98. The discrepancy between these performance values is explained by the fact that since FPs are misclassified features (e.g, numbers such as zeroes and nines, culverts, roundabouts), they can occur anywhere in the map. Because there are substantially higher numbers of such features across the maps relative to the number of wells, typically the FPs are located further than our buffer distance of 100 m from any documented well and are hence classified as UOWs. This results in a bias in most FPs being identified as potential UOWs, leading to a lower RVU compared to the U-Net model precision. Using the test precision of 0.98, the expected number of FPs is equal to 0.02 × *D*, where *D* is the number of detected wells. In comparison, the number of vetted potential UOWs is 4.3% of *D* (Table 1). Thus, assuming that all FPs are initially considered UOWs, the RVU is equal to 0.043 × *D*/(0.02 × *D* + 0.043 × *D*) = 0.68, which is comparable with the experimental average of 0.71 computed from the values in Table 1. This explains why the RVU can be lower than the performance expected from the algorithmic precision of 0.98, and even as low as 0.3 as in the case of Los Angeles County (see Figures SI10 and SI11 for additional details).

### Scaling and Transferability of Methodology to Other Regions

To our knowledge, our workflow is the first method that identifies potential UOWs at regional (county-level) spatial scales. The application of our workflow to larger spatial scales is possible due to the implementation of a deep learning computer vision algorithm on consistent maps available across the US, and our novel approaches for generating training labels, training the neural network, and vetting the results.

Traditional labeling for supervised semantic segmentation requires precisely labeling each pixel of the object of interest. This allows accurate identification of the exact boundaries of the object during training. Since our goal was to identify the location of the well symbols rather than their exact contour, we chose to label only the center of each well symbol to alleviate the burden of manually labeling individual pixels for each well to significantly speed up the labeling process. Due to the circular symmetry of the symbol, we generated a mask by choosing a radius consistent with the well symbol size. Also, we leveraged the convolutional nature of the U-Net algorithm, which aggregates the information on neighboring pixels. In this way the mask at the center of each well includes information about the surrounding symbol. For this reason we choose to generate pixel areas with values 1 that are entirely contained within the well symbols. In this way we were able to rapidly label 11,046 well symbols across 79 maps in a few days.

We also leveraged the concept of transfer learning, which refers to the use of previously learned knowledge in a related task, using it twice in our workflow. First, we use a U-Net model that is pretrained to classify images from the Imagenet database.^51^ This allows us to initialize the model with weights trained to extract generic and transferable features such as edges and shapes from millions of images that is then fine-tuned to our smaller map-based training set. Second, we trained our model solely on 79 maps from various regions in California (including Kern and LA counties), including maps with a wide variety of color distortions and land cover types, and used the trained algorithm to detect wells in both California and Oklahoma. Remarkably, the performance of the workflow measured by the RVU is higher in Oklahoma than in California. This shows the success of the transfer learning and potential extensibility of the method to new areas that the algorithm was not trained on. Upon visual inspection, we find the Oklahoma maps were more uniform (i.e, containing less variation in color palettes and color distortion) in comparison to the maps in California. Notably,as described above, the performance in Los Angeles County was poor (30% vetted to unvetted potential UOWs) likely due to the larger number of confounding features present in urban areas, such as roundabouts and cul-desacs. In rural areas the most common causes of misclassification are numbers and letters containing circular patterns, like the number “9” and the letter “o”, and hilltops, denoted by quasi-circular topographic lines.

Additionally, we developed a novel approach to vetting potential UOWs with a custom script that allowed screening of hundreds of images in a short time. For each unvetted potential UOW detected by the computer vision algorithm, the script displays the relevant part of the map cropped and enlarged in an interactive window. An operator visually assesses the detection and confirms or rejects it with a simple mouse click. Using our script, we are able to vet approximately 1000 potential UOWs in 1 h of manual inspection.

## Data Availability

The model used, and data from the results of this study is made publicly available through the U.S. Department of Energys Energy Data Exchange (EDX) at the url 10.18141/2452768. The geographical coordinates of the potential UOWs as well as the satellite and field verified UOWs will be part of the data set, and also can be found in the Supporting Information and in the Public CATALOG Data Dashboard at the following url: https://arcgis.netl.doe.gov/portal/apps/experiencebuilder/experience/?id=845a0643bbc64b0dba52be0016293f74&page=page_3. The data used as inputs in our workflow are publicly available, and should be downloaded from the original data sources. The USGS HTMC maps can be downloaded from the National Geologic Map Database project webpage https://ngmdb.usgs.gov/topoview/.^31^ The official California and Oklahoma and Osage Nation O&G well databases can be found at https://www.conservation.ca.gov/calgem/maps/Pages/GISMapping2.aspx,^33^https://gisdata-occokc.opendata.arcgis.com/^34^ and https://www.osageminerals.org/(35) respectively.

## Supporting Information Available

Potential UOWs locations found with our workflow (ZIP)
Visible potential UOWs from our workflow and their exact location from satellite imagery (ZIP)
Additional experimental details, materials, and methods, including plots and tables (PDF)

## Notes

Expansion of our approach to other regions will require addressing some of the issues described above, including potentially increasing the size of the training data set, particularly for urban areas, and exploration of other image segmentation architectures to improve precision. However, our demonstration of the methodology for the study areas, combined with the continental coverage of the HTMC maps indicates the potential of scaling and transferring our workflow to other O&G producing regions of the US, and will allow stakeholders to rapidly identify UOWs in specific areas relevant to their interests.
