# Supplementary material for: A Deep Learning Based Framework to Identify Undocumented Orphaned Oil and Gas Wells from Historical Maps: A Case Study for California and Oklahoma
Source: Environ Sci Technol. 2024 Dec 4;58(50):22194–203. doi: 10.1021/acs.est.4c04413 (PMC11656717; doi:10.1021/acs.est.4c04413)
Supplement: Supplementary file 3 — es4c04413_si_003.pdf [file es4c04413_si_003.pdf]

Supporting Information
A Deep Learning Based Framework to Identify Undocumented Orphaned Oil and Gas Wells from Historical Maps: a Case Study for California and Oklahoma

Fabio Ciulla,* Andre Santos, Preston Jordan, Timothy Kneafsey, Sebastien C. Biraud, and Charuleka Varadharajan*

Earth and Environmental Sciences Area, Lawrence Berkeley National Laboratory, Berkeley, CA 94720, United States

E-mail: fciulla@lbl.gov; cvaradharajan@lbl.gov

Summary: 25 pages, 11 figures, 2 tables

List of Figures

SI1 Examples of 1000x1000 pixel tiles from HTMC maps showing a diversity of backgrounds and discolorations a) undefined background displayed in white, b) undefined background with color distortion where the white background has become yellow with age, c) very dense topographic lines on yellow undefined background, d) green background, indicating vegetation, in solid and dotted patterns, e) blue background indicating a water body, f) red background indicating urban area, g) purple features, mostly oil wells and roads, on undefined background denoting a change in the landscape, and h) examples of cultural features such as highways, buildings and a golf course. S6

SI2 Oil production in different states of the US from 1876 to 1935 a) annually and b) cumulatively. S7

SI3 Oil production in different counties of California from 1876 to 1915 a) annually and b) cumulatively. S7

SI4 Examples of images produced by the vetting script algorithm to verify symbol detection. Each square shows the area surrounding an unvetted potential UOW, identified with a red circle and a blue well identification number. a) Subset of unvetted potential UOWs in Venice, Los Angeles County, CA. The unvetted potential UOWs with IDs 1 and 2 are discarded because they are a cul-de-sac and the letter “d” respectively. b) Subset of unvetted potential UOWs in Avant, Osage County, OK. The unvetted potential UOW with ID 8 is the number “9” and so discarded. S9

SI5 Six examples of satellite images displaying evidence of lifting equipment in proximity to detected UOWs. The exact locations of the UOWs as identified by the computer vision algorithm are displayed as red markers. The letters a-f identifying each image corresponds to the superscripts of the unique site IDs in Table SI1. . . . . S11

SI6 Graphical representation of four magnetic surveys taken in proximity of potential UOWs sites. Red circles are the exact coordinates produced by the computer vision algorithm. The dots represent the data collected in the field, whose location is generated by the magpack's GPS, and the color indicating the total magnetic field as displayed in the respective color bars. The background is a 2D linear interpolation of the magnetic field from the dots, whose color is coherent with the color bar, and is intended as a visual aid to visualize the magnetic field anomaly. The letters a-d identifying each image correspond to the superscripts of the unique site IDs in Table SI2. . . . . S16
SI7 Flowchart displaying the workflow followed for magnetic surveys in the field. S18
SI8 Comparison between image features extraction on the same map tiles using a combination of traditional computer vision approaches like edge detection, color clustering and template matching (a, b) vs. deep learning U-Net architecture (c, d). Detected circles are highlighted by green discs in (a) and (b) and by blue circles in (c) and (d). Note how the identification of purple circles on the top left of (a) and the top of (b) is mistakenly missing. Also, in (a) parts of a dirt road (two parallel black dashed lines) and a railroad (continuous black lines) in the middle of the images have been misclassified as circles, as well as some alphanumerical characters. The corresponding areas in (c, d) display correct classification. S20
SI9 Values of loss (a) and IoU score (b) for training, in yellow and validation (in red) sets. In c) each datapoint (blue dots) displays precision and recall for the validation set when the area of the disc for detection is set to the number shown close to each point. The red dot refers to precision and recall for the test set. S22

SI10 Pictorial representation of the difference in detection performance between all symbols detected by the model, and UOWs only as a Venn diagram. a) The set of all detected symbols is shown in blue. The set of false positives (FP) are a subset displayed in orange. The set of unvetted UOWs (shown in red) is composed of correctly detected symbols that are UOWs (i.e. hollow circles that are further than 100 m from the closest documented wells). The set of vetted UOWs is the difference between unvetted UOWs and false UOWs. The set of false UOWs are FPs that are considered UOWs because their distance to documented wells is greater than 100 m. As explained in the Discussion, most FPs tend to be classified as UOWs because the incorrectly detected features tend to be greater than 100 m from documented wells b) The algorithmic precision of our U-Net model is the ratio between true positives (TP) and all detected symbols (TP+FP), equal to 0.98. c) The RVU, is computed as the ratio between the vetted and the unvetted UOWs. The large overlap between the FP, and the unvetted UOWs lead to an average RVU of 0.71 in our areas of investigation. S23
SI11 Examples of wrongly detected well symbols that have been labeled as UOWs from Beverly Hills, California. Specifically a) a numerical value, b) a letter symbol, c-e) cul-de-sacs on different backgrounds and f) a rotatory, are mistakenly detected as well symbols. All of these FPs are considered UOWs because of their distance to the closest documented wells (the brown numbers in each figure represent this distance in meters). S24

List of Tables

SI1 List of potential UOW sites in Osage County, OK where an oil rig is visible from satellite images. Sites are identified by the union of the name of the map where a UOW has been detected, and a numerical id unique per each map. The superscript letters shown for 6 unique IDs match the images in Figure SI5. S11

SI2 List of 35 potential UOW sites in Kern and Osage counties that were visited in multiple field campaigns. Of the 27 that could be accessed, 15 were verified as UOWs. For each, the distance between the location as detected by the computer vision algorithm and the maximum anomaly from the magnetic survey is given. Sites are identified by the union of the name of the map where a UOW has been detected, and a numerical id unique per each map. The sites with superscript letters correspond to the ones whose magnetic surveys are displayed in Figure SI6. . . . . S15

1 Historical Maps Dataset

The maps in the HTMC dataset have a range of background colors that indicate land surface cover (Figure SI1), and topographic contour lines, typically shown in brown. Approximately 200 symbols in different colors indicate natural features like mountains, rivers and vegetation, and manmade features like roads, buildings, and industrial structures.[1]

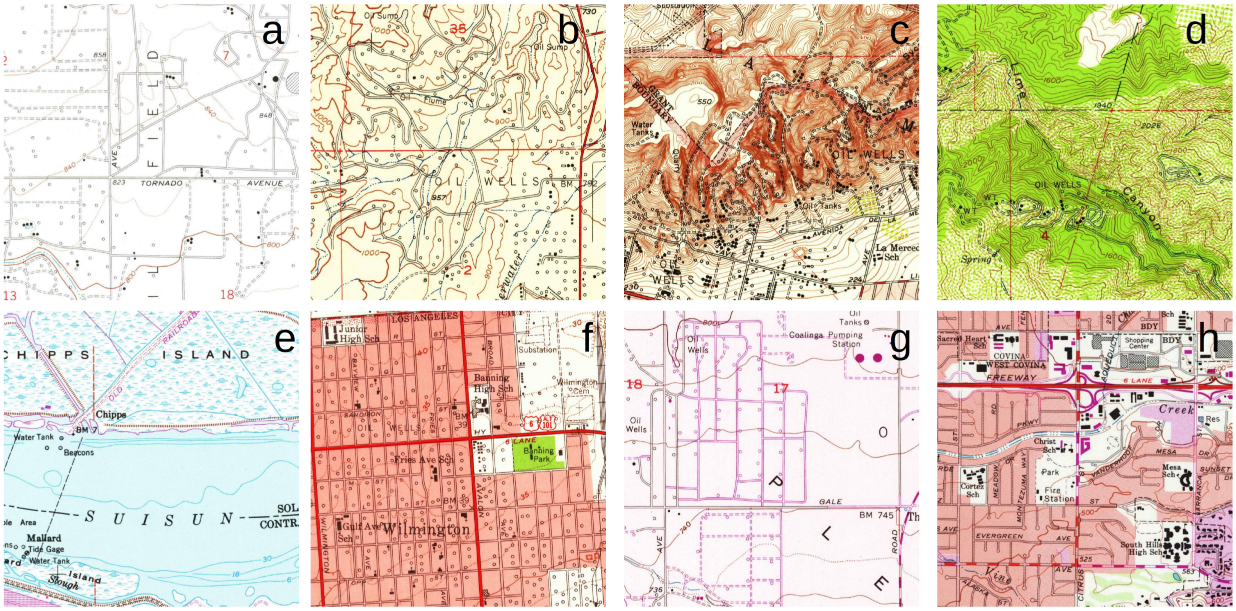

Figure SI1: Examples of 1000x1000 pixel tiles from HTMC maps showing a diversity of backgrounds and discolorations a) undefined background displayed in white, b) undefined background with color distortion where the white background has become yellow with age, c) very dense topographic lines on yellow undefined background, d) green background, indicating vegetation, in solid and dotted patterns, e) blue background indicating a water body, f) red background indicating urban area, g) purple features, mostly oil wells and roads, on undefined background denoting a change in the landscape, and h) examples of cultural features such as highways, buildings and a golf course.

2 Early Production Data

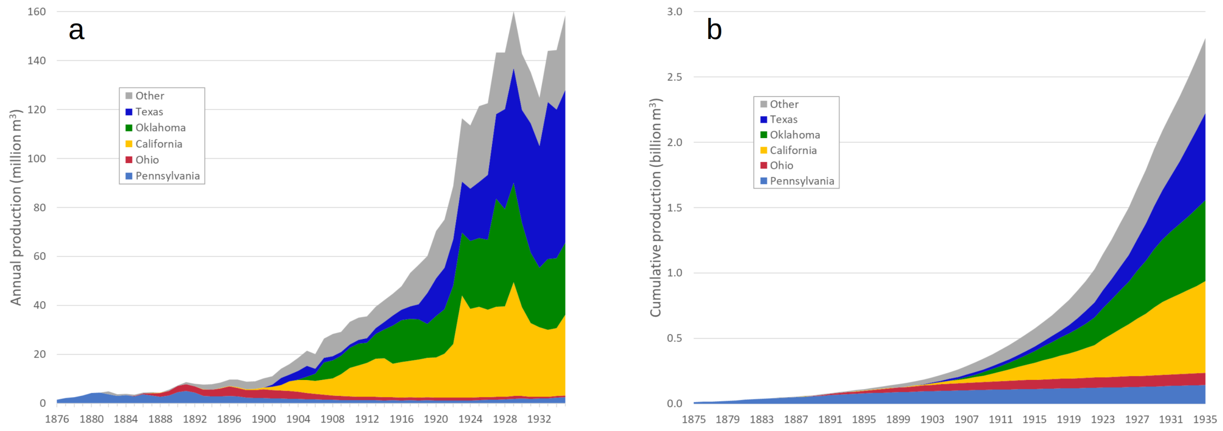

Figure SI2: Oil production in different states of the US from 1876 to 1935 a) annually and b) cumulatively.

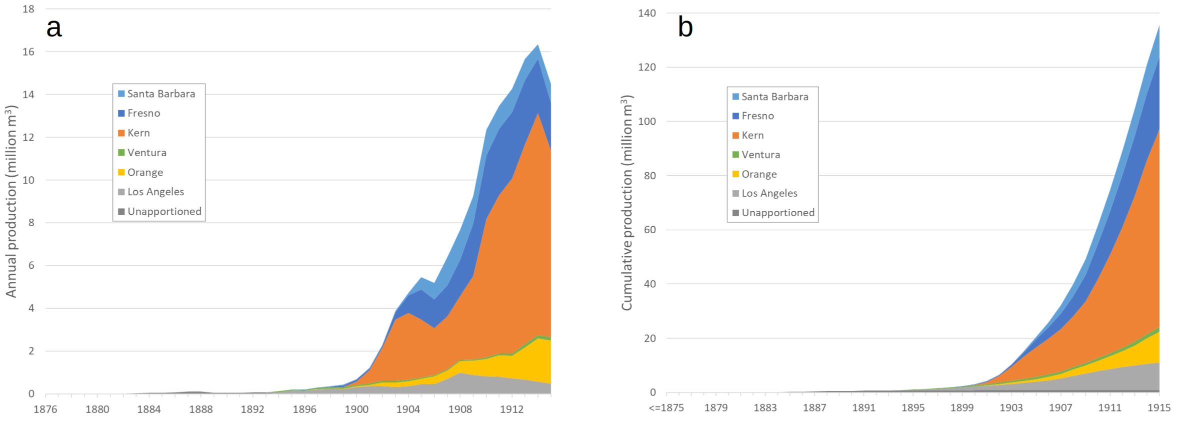

Figure SI3: Oil production in different counties of California from 1876 to 1915 a) annually and b) cumulatively.

3 Custom Vetting Script

Machine learning algorithms produce a certain number of false positives, namely wrongly detected objects. In our case these are symbols or other graphical patterns that are not black circles - the symbol for O&G wells - but are erroneously detected as such. To visually check every detected object would be extremely lengthy, given that the number of wells in a county can be 10,000 or more. Besides, it would defeat the purpose of having a scalable machine learning framework for detection. However, because the targets of our study are UOWs, which represent a small ratio of the overall detected wells, we can restrict the manual inspection only to those. For this purpose we developed a script that crops the areas surrounding each detected potential UOW and conveniently displays them for human verification (Figure SI4). The images are interactive, and an operator can validate or discard any detected potential UOW with a simple mouse click. In this way we are able to validate about 1,000 potential UOWs per hour. The ability to remove falsely detected symbols is especially beneficial when potential UOWs are selected for a field campaign, in that it takes only a few seconds to discard a wrongly detected well remotely, potentially saving hours in the field by preventing visiting a wrong site.

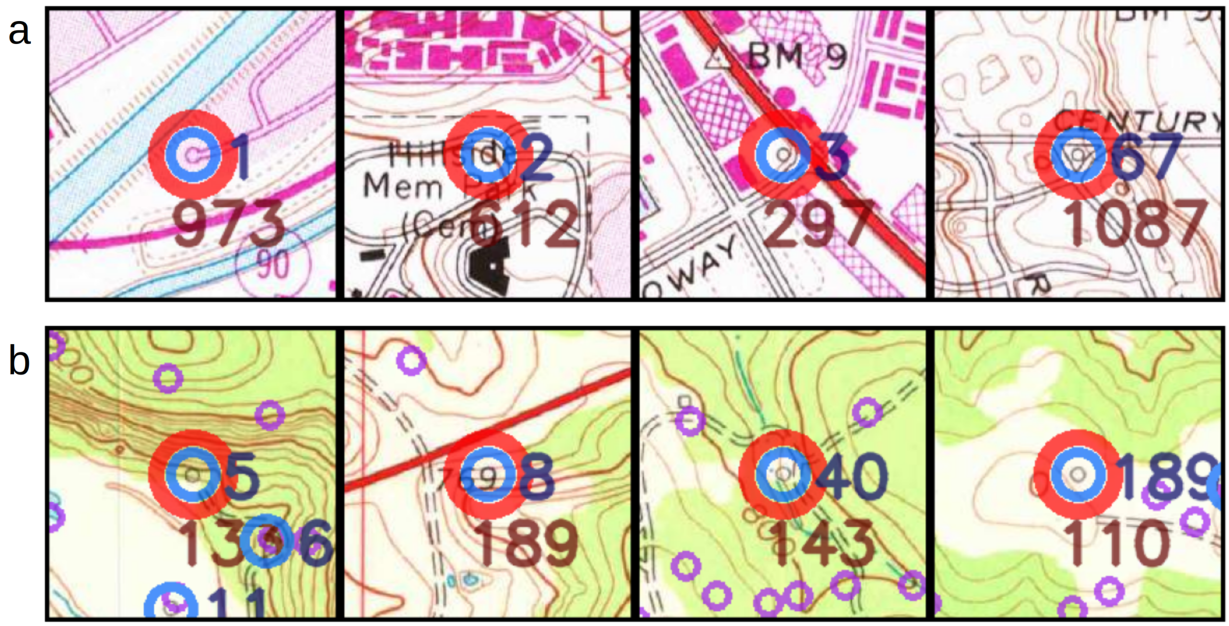

Figure SI4: Examples of images produced by the vetting script algorithm to verify symbol detection. Each square shows the area surrounding an unvetted potential UOW, identified with a red circle and a blue well identification number. a) Subset of unvetted potential UOWs in Venice, Los Angeles County, CA. The unvetted potential UOWs with IDs 1 and 2 are discarded because they are a cul-de-sac and the letter “d” respectively. b) Subset of unvetted potential UOWs in Avant, Osage County, OK. The unvetted potential UOW with ID 8 is the number “9” and so discarded.

4 UOWs Confirmation with Satellite Images

Unique ID	Coordinates from Algorithm	Coordinates from Satellite	Algorithm to Satellite Distance (m)
Whippoorwill_114[a]	36.952074, −96.150396	36.952087, −96.1505	9.4
Wolco_267[b]	36.547528, −96.053786	36.547565, −96.053836	6.1
Wolco_330	36.536064, −96.067767	36.536221, −96.067732	17.7
Wolco_337	36.533794, −96.064703	36.533795, −96.064623	7.1
Sand Springs_171[c]	36.221682, −96.049544	36.221728, −96.04956	5.4
Sand Springs_197	36.212889, −96.043567	36.212909, −96.04359	3.0
Sand Springs_207	36.21163, −96.040744	36.211649, −96.040778	3.7
Sand Springs_208	36.211508, −96.043237	36.211585, −96.043275	9.3
Sand Springs_213	36.210752, −96.033306	36.210806, −96.033391	9.7
Avant SE_145[d]	36.271765, −96.054942	36.271755, −96.0549	3.9
Avant SE_250	36.254916, −96.061559	36.254905, −96.061368	17.2
Barnsdall_86[e]	36.574118, −96.240272	36.57411, −96.240353	7.3
Wynona_88[f]	36.594351, −96.351485	36.594307, −96.351524	6.1
Wynona_85	36.596429, −96.353258	36.596513, −96.353329	11.3
Burbank_391	36.668958, −96.659221	36.668952, −96.659331	9.9
Burbank_525	36.640574, −96.655535	36.640649, −96.655667	14.5
Avant NW_20	36.487207, −96.175554	36.487228, −96.175547	2.4
Avant NW_59	36.443436, −96.195222	36.44329, −96.195306	17.9
Shidler_28	36.840984, −96.719643	36.840892, −96.719728	12.7
Woolaroc_52	36.719663, −96.064936	36.719649, −96.065019	7.6

Hominy NE_40	36.465479, −96.26116	36.46538, −96.261211	11.8
Hominy NE_96	36.422597, −96.337448	36.422718, −96.337337	16.8
Hominy NE_116	36.379709, −96.334493	36.379756, −96.334631	13.4
Bowring SE_124	36.79938, −96.090287	36.799331, −96.090252	6.3
Bowring SE_191	36.783122, −96.084818	36.78311, −96.084748	6.3
Hominy_15	36.494148, −96.440541	36.494143, −96.440611	6.3
Lucy Creek_208	36.55178, −96.503799	36.551834, −96.503665	13.4
New Prue_70	36.286482, −96.327898	36.286452, −96.32805	14.0
Wekiwa_37	36.191651, −96.203987	36.191633, −96.204019	3.5

Table SI1: List of potential UOW sites in Osage County, OK where an oil rig is visible from satellite images. Sites are identified by the union of the name of the map where a UOW has been detected, and a numerical id unique per each map. The superscript letters shown for 6 unique IDs match the images in Figure SI5.

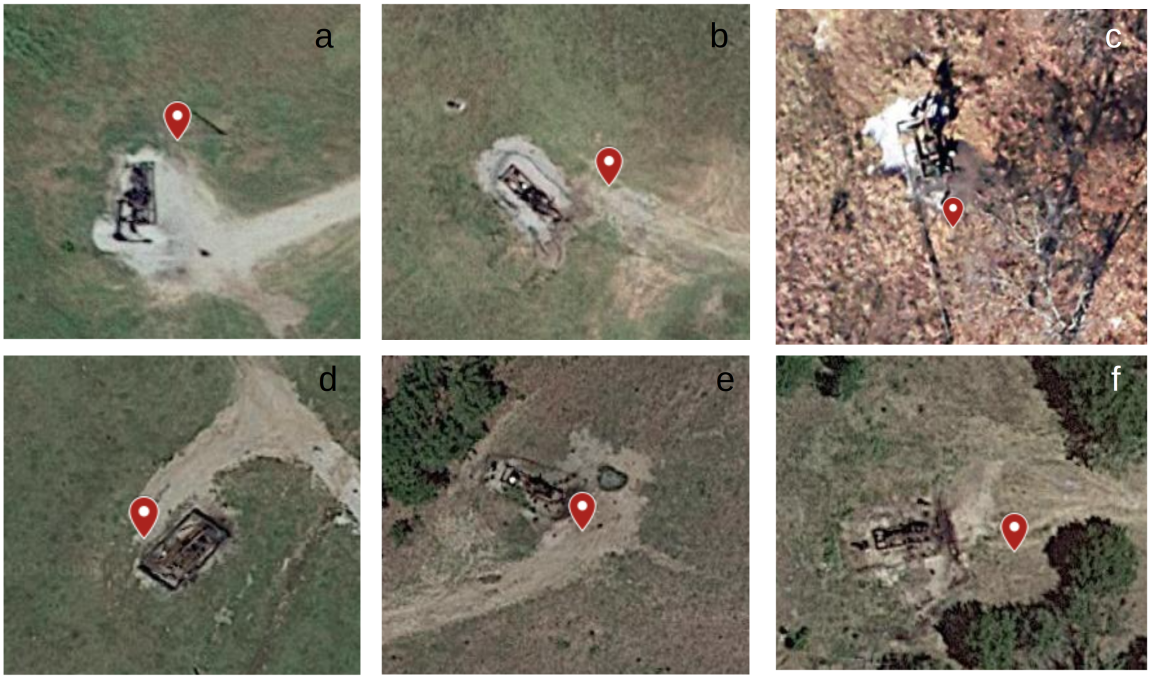

Figure SI5: Six examples of satellite images displaying evidence of lifting equipment in proximity to detected UOWs. The exact locations of the UOWs as identified by the computer vision algorithm are displayed as red markers. The letters a-f identifying each image corresponds to the superscripts of the unique site IDs in Table SI1.

5 UOWs Confirmation in the Field Investigations

Country	Unique ID	Field Campaign Date	Coordinates from Algorithm	Coordinates from Field Investigation	Algorithm to Field Distance (m)
Kern, CA	Belridge_855	2/29/2024	35.404193, −119.726902	35.404135, −119.726897	6.47
Kern, CA	Belridge_935	2/29/2024	35.390873, −119.739353	35.390804, −119.739419	9.76
Kern, CA	Mouth of Kern_4	6/25/2023	35.247267, −119.29873	No Access	N/A
Kern, CA	Mouth of Kern_7	6/25/2023	35.246478, −119.29096	No Access	N/A
Kern, CA	Mouth of Kern_27	2/29/2024	35.237785, −119.256471	Not Found	N/A
Kern, CA	Mouth of Kern_31	3/1/2024	35.23597, −119.25366	Not Found	N/A
Kern, CA	Mouth of Kern_32	3/1/2024	35.174029, −119.372401	Not Found	N/A
Kern, CA	Mouth of Kern_46[a]	3/1/2024	35.160292, −119.371744	35.160441, −119.371867	20.01
Kern, CA	Oildale_944	6/25/2023	35.455986, −119.026593	No Access	N/A
Kern, CA	Oildale_1531	6/25/2023	35.376706, −119.052258	No Access	N/A

Kern, CA	Reward_362	6/25/2023	35.30938, −119.711617	No Access	N/A
Kern, CA	Reward_407	3/1/2024	35.289344, −119.66002	Not Found	N/A
Kern, CA	Taft_791	3/1/2024	35.135374, −119.491474	35.135336, −119.491479	4.25
Kern, CA	Taft_550	3/1/2024	35.163154, −119.418854	35.163299, −119.418833	16.29
Kern, CA	Taft_572	3/1/2024	35.160947, −119.491117	35.160870, −119.491098	8.73
Kern, CA	Taft_611	3/1/2024	35.157431, −119.488483	35.157572, −119.488446	16.14
Kern, CA	Taft_624[b]	3/1/2024	35.155554, −119.424788	35.155541, −119.424787	1.50
Kern, CA	Tupman_171	6/25/2023	35.275825, −119.298932	No Access	N/A
Kern, CA	Tupman_187	6/25/2023	35.274404, −119.262884	No Access	N/A
Kern, CA	Tupman_284	6/25/2023	35.264725, −119.294713	No Access	N/A
Kern, CA	West Elk Hills_89	3/1/2024	35.250878, −119.607658	35.251037, −119.607747	19.48
Osage, OK	Pearsonia NE_27	3/12/2024	36.981853, −96.358322	Not Found	N/A
Osage, OK	Pearsonia NE_16	3/12/2024	36.989176, −96.356858	Not Found	N/A

Osage, OK	Pearsonia NE_6	3/12/2024	36.994767, −96.361495	Not Found	N/A
Osage, OK	Pearsonia NE_5	3/12/2024	36.996514, −96.362345	Not Found	N/A
Osage, OK	Pearsonia NE_4	3/12/2024	36.996828, −96.360418	Not Found	N/A
Osage, OK	Pearsonia NE_3	3/12/2024	36.997509, −96.360350	Not Found	N/A
Osage, OK	Pearsonia NE_47	3/12/2024	36.930000, −96.373669	Not Found	N/A
Osage, OK	Pearsonia NE_52	3/12/2024	36.928270, −96.374768	Not Found	N/A
Osage, OK	Barnsdall_199	3/14/2024	36.531853, −96.135149	36.531873, −96.135135	2.59
Osage, OK	Wolco_270	3/14/2024	36.547304, −96.085379	36.547347, −96.085409	5.51
Osage, OK	Wolco_277[c]	3/14/2024	36.546481, −96.080000	36.546413, −96.080093	11.28
Osage, OK	Wolco_287	3/14/2024	36.544853, −96.070354	36.544982, −96.070382	14.53
Osage, OK	Wolco_321[d]	3/14/2024	36.537895, −96.068629	36.538106, −96.068571	24.02
Osage, OK	Wolco_330	3/14/2024	36.536064, −96.067767	36.536203, −96.067748	15.51

Table SI2: List of 35 potential UOW sites in Kern and Osage counties that were visited in multiple field campaigns. Of the 27 that could be accessed, 15 were verified as UOWs. For each, the distance between the location as detected by the computer vision algorithm and the maximum anomaly from the magnetic survey is given. Sites are identified by the union of the name of the map where a UOW has been detected, and a numerical id unique per each map. The sites with superscript letters correspond to the ones whose magnetic surveys are displayed in Figure SI6.

6 Magnetic Survey Examples

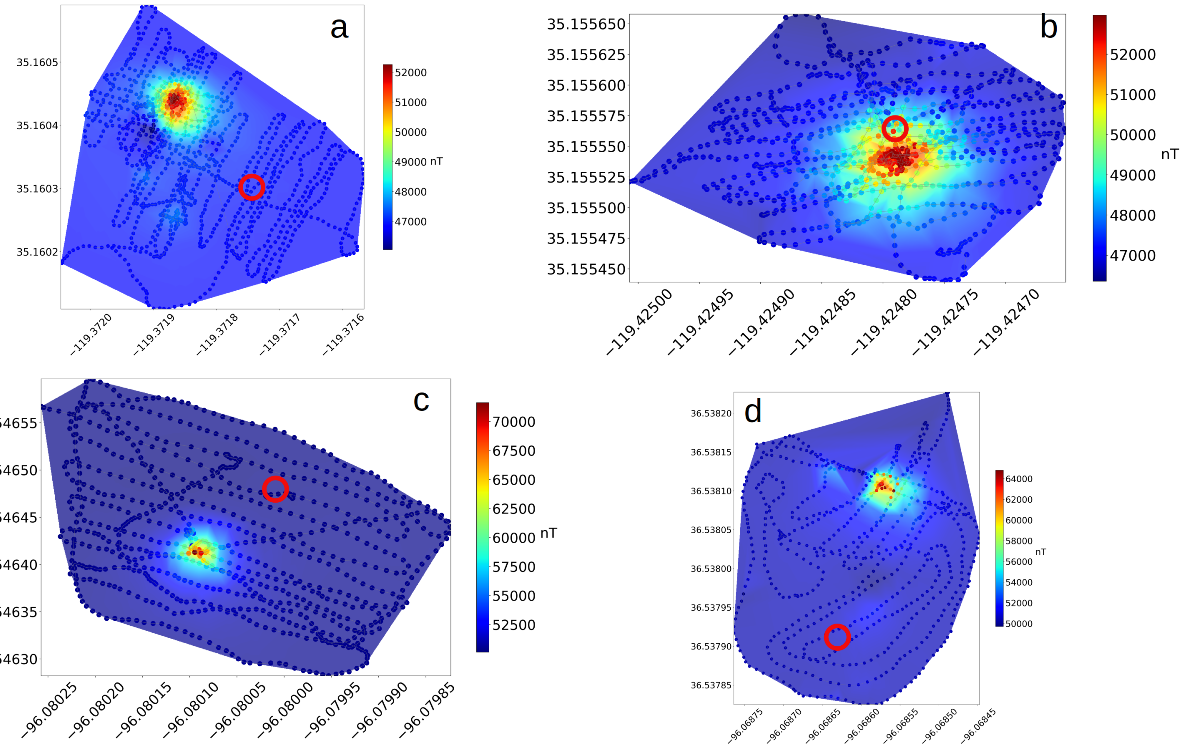

Figure SI6: Graphical representation of four magnetic surveys taken in proximity of potential UOWs sites. Red circles are the exact coordinates produced by the computer vision algorithm. The dots represent the data collected in the field, whose location is generated by the magpack's GPS, and the color indicating the total magnetic field as displayed in the respective color bars. The background is a 2D linear interpolation of the magnetic field from the dots, whose color is coherent with the color bar, and is intended as a visual aid to visualize the magnetic field anomaly. The letters a-d identifying each image correspond to the superscripts of the unique site IDs in Table SI2.

7 Field Data Collection Workflow

The field data collection workflow was as follows. Upon arrival at the field site, if a structure was visible (e.g., well head, oil rig, concrete pad) in the immediate vicinity of the potential UOW coordinates, the equipment operator walked in a gridded pattern to generate a square 50 m long on each side centered around the structure. If no structure was visible, the size of the square was extended to 100 m per side. The exploration pattern at times deviated from the grid due to challenging terrain.

The presence of a buried vertical metal pipe, such as a well, produces a magnetic anomaly that depends on its orientation, overall length, material and distance from the surface.[2,3] Background magnetic levels and uncertainty are retrieved from the NOAA World Magnetic Model (WMM) for each location. Magnetic anomalies greater than the background plus three times the uncertainty and with approximate circular symmetry (in literature often referred to as a bulls-eye) can be generated by a long vertical metal pipe below ground and so was considered evidence for the presence of a buried well.[4] If no anomaly was detected, the operator actively searched for magnetic anomalies leveraging the instrument's real-time measurement capability and, if found, proceeded with the gridded search 50 m on each side as described above around the highest value of the discovered magnetic anomaly (Figure SI7).

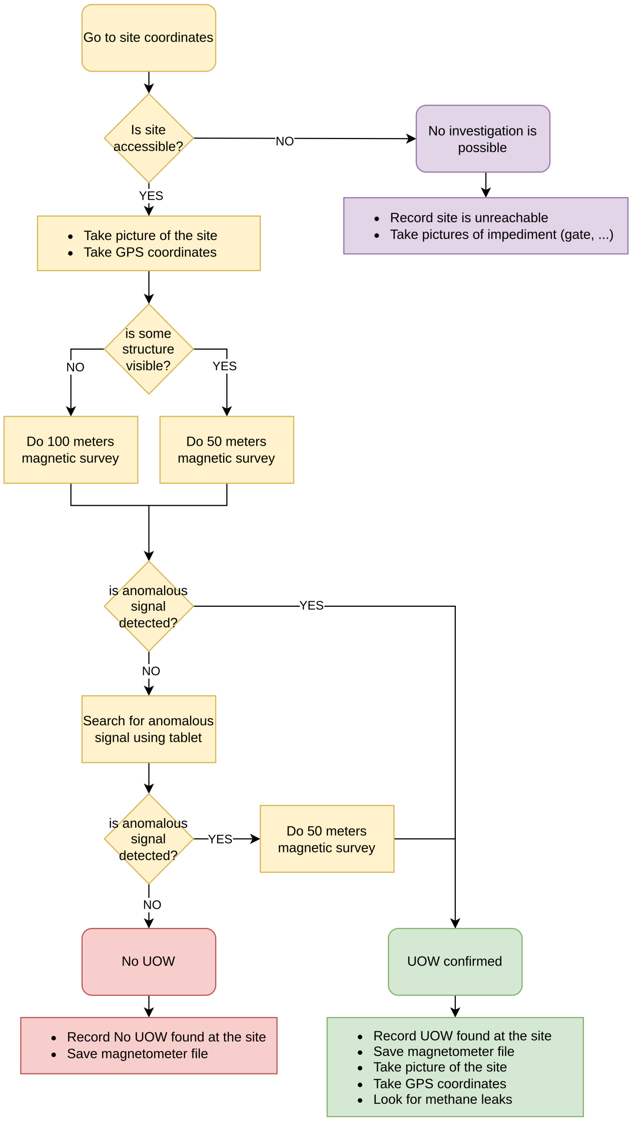

Figure SI7: Flowchart displaying the workflow followed for magnetic surveys in the field.

8 Comparison between Traditional Computer Vision Techniques and U-Net Semantic Segmentation

The background color of the historical maps plays an important role when trying to detect well symbols using traditional computer vision techniques. For example, background colors affect the image preprocessing step of color quantization that is used to separate colors in the maps and isolate color-dependent features. The high sensitivity of this approach to parameter choice precludes the use of an unique set of parameters across maps with different backgrounds and color distortions, and impedes model generalizability. We investigated the overall performance of traditional techniques, including a combination of blurring, color quantization edge detection, and template matching, and found them to be unsatisfactory (Figure SI8).

Since we used a U-Net neural network architecture pretrained on the ImageNet dataset, we adopted the same preprocessing of inputs that were used in the pretraining. In the Tensorflow implementation that we used, the preprocessing step corresponds to an identity function, where the image is provided without modifications to the model as an RGB tensor of integers ranging from 0 to 255. No other preprocessing steps are adopted.

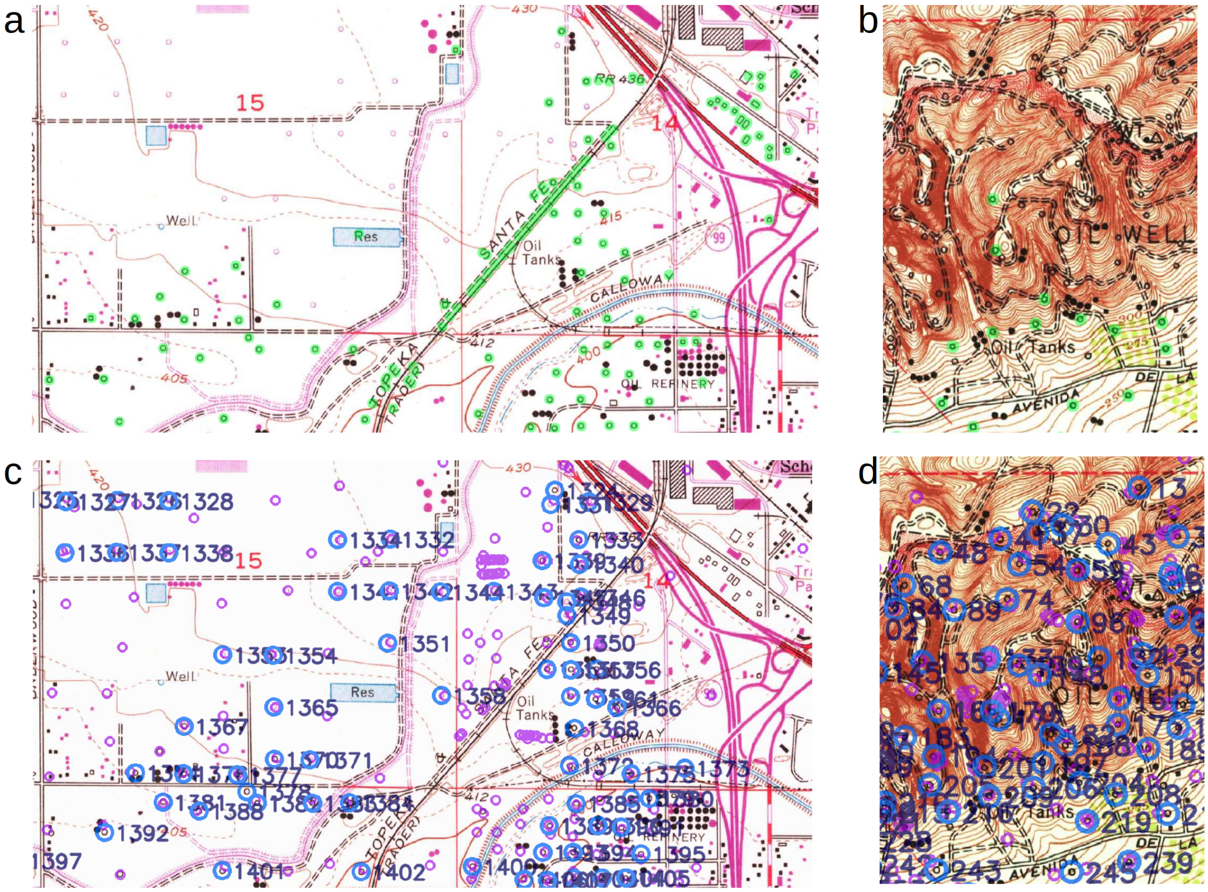

Figure SI8: Comparison between image features extraction on the same map tiles using a combination of traditional computer vision approaches like edge detection, color clustering and template matching (a, b) vs. deep learning U-Net architecture (c, d). Detected circles are highlighted by green discs in (a) and (b) and by blue circles in (c) and (d). Note how the identification of purple circles on the top left of (a) and the top of (b) is mistakenly missing. Also, in (a) parts of a dirt road (two parallel black dashed lines) and a railroad (continuous black lines) in the middle of the images have been misclassified as circles, as well as some alphanumerical characters. The corresponding areas in (c, d) display correct classification.

9 U-Net Architecture

The U-Net neural network adopetd in this study uses the Resnet-34 architecture[5] for image segmentation on the image mask pairs as implemented in the Segmentation Models github repository.[6] The model has 199 layers and 24,438,804 trainable parameters, and is initialized with weights pretrained on the Imagenet database.[7] We also investigated the Resnet-50 architecture and found no substantial increase in model performance. Consequently we used the model with less parameters to reduce the computational complexity and expense.

The binary focal loss[8] was chosen as a loss function due to its ability to focus on parts of the images that are hard to classify. We used the parameters specified in the original paper,[8] including the threshold value of 0.5. We explored different threshold values of the loss function for mapping pixels into zeroes and ones. Values lower than 0.5 resulted in more pixels being detected as targets, reducing precision and increasing recall. Conversely, values higher than 0.5 resulted in fewer pixels being detected as targets, increasing precision and reducing recall. Since the final goal of the algorithm is to detect the well symbols as a whole, we aggregated adjacent detected pixels into one object (as described in section Segmentation Post-Processing in main manuscript Methods). We noticed that applying a threshold on the areas of the aggregated pixels (referred to as area threshold) produced the same effect in classifying detected objects as tuning the threshold value of the loss function. For this reason, we kept the loss function threshold to the default 0.5 value[8] and instead, chose to fine-tune the area threshold. When using 45 pixels as an area threshold for the detected object (i.e. considered a true positive), the precision and recall were 0.994 and 0.904 respectively for the training set, 0.993 and 0.885 for the validation set, and 0.982 and 0.882 for the test set.

We implemented the Adam optimizer as a gradient descent algorithm as it is more flexible for parameter choice compared to traditional optimizers like the Stochastic Gradient Descent (SGD) with momentum.[9] We explored model performance with different learning rates of 1e-5, 5e-5, 1e-4 and 1e-3 explored, and chose the value of 5e-5, which had the best performance. The sigmoid function was used as the final activation function.

The training set was split into batches of 8 images each and the learning was performed for 30 epochs. The model for which the intersection over union (IoU) metric reaches a plateau on the validation set and starts to overfit on the training set was saved and used for prediction. This happened at epoch 10, when the IoU for the validation set is equal to 0.76 (Figure SI9). The area of one disc with radius 4 pixels, used in the mask to denote the targets, is equal to 49 pixels.

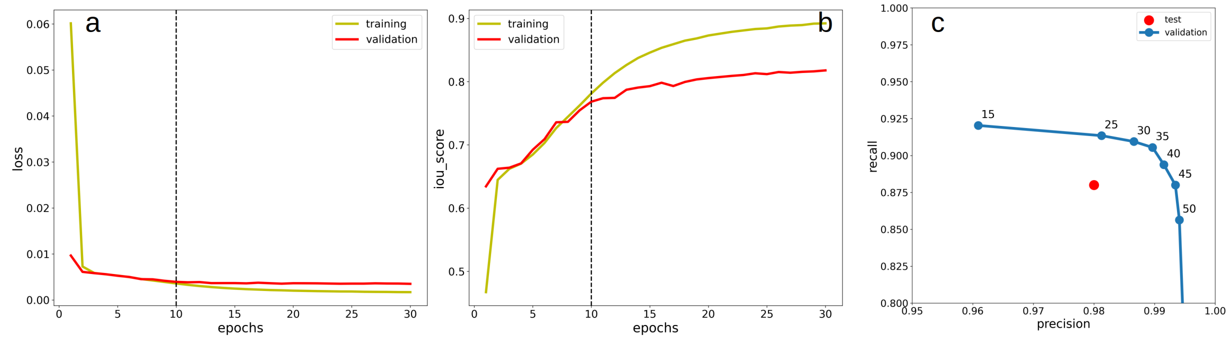

Figure SI9: Values of loss (a) and IoU score (b) for training, in yellow and validation (in red) sets. In c) each datapoint (blue dots) displays precision and recall for the validation set when the area of the disc for detection is set to the number shown close to each point. The red dot refers to precision and recall for the test set.

10 Explanation of the Difference between Model Precision and the Ratio of Vetted to Unvetted UOWs (RVU)

Here, we further explain the discrepancy between algorithmic performance and ratio of vetted to unvetted UOWs (RVUs), using Venn diagrams as a visual aid (Figure SI10).

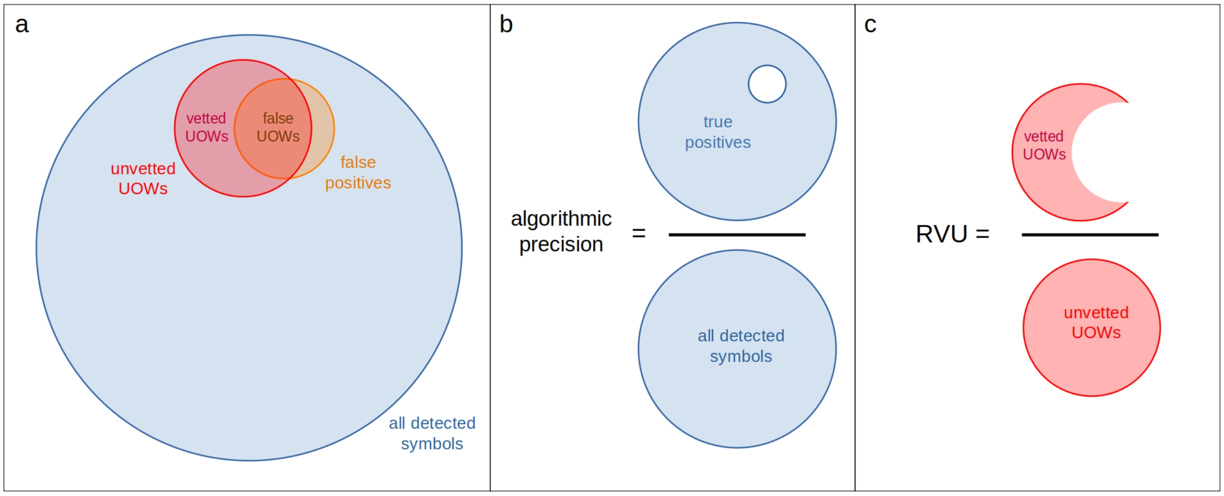

Figure SI10: Pictorial representation of the difference in detection performance between all symbols detected by the model, and UOWs only as a Venn diagram. a) The set of all detected symbols is shown in blue. The set of false positives (FP) are a subset displayed in orange. The set of unvetted UOWs (shown in red) is composed of correctly detected symbols that are UOWs (i.e. hollow circles that are further than 100 m from the closest documented wells). The set of vetted UOWs is the difference between unvetted UOWs and false UOWs. The set of false UOWs are FPs that are considered UOWs because their distance to documented wells is greater than 100 m. As explained in the Discussion, most FPs tend to be classified as UOWs because the incorrectly detected features tend to be greater than 100 m from documented wells b) The algorithmic precision of our U-Net model is the ratio between true positives (TP) and all detected symbols (TP+FP), equal to 0.98. c) The RVU, is computed as the ratio between the vetted and the unvetted UOWs. The large overlap between the FP, and the unvetted UOWs lead to an average RVU of 0.71 in our areas of investigation.

The algorithmic precision is the ability of the computer vision algorithm to correctly recognize wells symbols, and is equal to 0.98 in the test set. The RVU, computed from the manual vetting of all the maps investigated averages, to 0.71. However, in Los Angeles

County the RVU is low (0.3), even though maps from the region were included in the training set. In contrast the RVU was higher in Oklahoma and Osage counties, even though no maps from Oklahoma were used for training purposes. This difference is because of confounding symbols such as rotatories and cul-de-sacs that tend to be present in urban environments (Figure SI11).

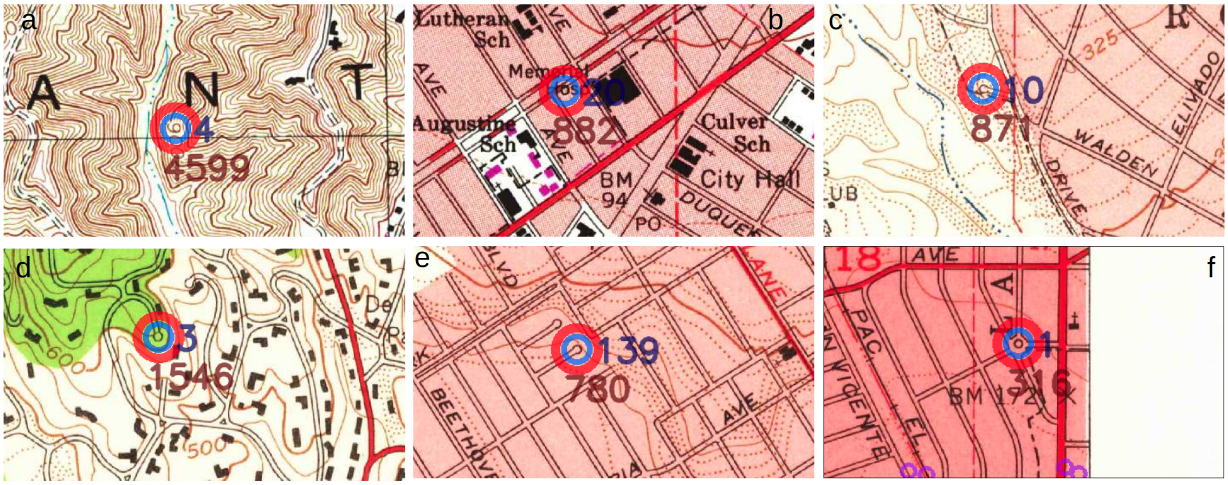

Figure SI11: Examples of wrongly detected well symbols that have been labeled as UOWs from Beverly Hills, California. Specifically a) a numerical value, b) a letter symbol, c-e) cul-de-sacs on different backgrounds and f) a rotatory, are mistakenly detected as well symbols. All of these FPs are considered UOWs because of their distance to the closest documented wells (the brown numbers in each figure represent this distance in meters).

References

(1) Aerials, H. United States Geologic Service (USGS) Topographic Map Key. https://www.historicaerials.com/topo-map-key, (accessed 2024-10-10).

(2) Singh, S. K.; Sabina, F. J. Magnetic anomaly due to a vertical right circular cylinder with arbitrary polarization. *Geophysics* **1978**, *43*, 173–178.

(3) McKenzie, K. B. The magnetic field and magnetic gradient tensor for a right circular cylinder. *Exploration Geophysics* **2022**, *53*, 329–358.

(4) Hammack, R. W.; Veloski, G. A.; Hodges, D. G.; White, C. M. Methods for Finding Legacy Wells in Large Areas. *NETL-TRS-6-2016; EPAct Technical Report Series* **2016**, 28.

(5) He, K.; Zhang, X.; Ren, S.; Sun, J. Deep Residual Learning for Image Recognition. *arXiv* **2015**,

(6) Iakubovskii, P. Segmentation Models. https://github.com/qubvel/segmentation_models, 2019.

(7) Deng, J.; Dong, W.; Socher, R.; Li, L.-J.; Li, K.; Fei-Fei, L. Imagenet: A large-scale hierarchical image database. 2009 IEEE conference on computer vision and pattern recognition. 2009; pp 248–255.

(8) Lin, T.-Y.; Goyal, P.; Girshick, R.; He, K.; Dollár, P. Focal Loss for Dense Object Detection. *arXiv* **2017**,

(9) Kingma, D. P.; Ba, J. Adam: A Method for Stochastic Optimization. *arXiv* **2014**,
